# Gene expression in murine mammary epithelial stem cell-like cells shows similarities to human breast cancer gene expression

**DOI:** 10.1186/bcr2256

**Published:** 2009-05-08

**Authors:** Cecilia Williams, Luisa Helguero, Karin Edvardsson, Lars-Arne Haldosén, Jan-Åke Gustafsson

**Affiliations:** 1 Department of Biology and Biochemistry, Center for Nuclear Receptors and Cell Signaling, University of Houston, 3013 Science & Engineering Research Center, Houston, TX 77204, USA; 2 Department of Biosciences and Nutrition, Novum, Karolinska Institutet, 141 57 Stockholm, Sweden

## Abstract

**Introduction:**

Mammary stem cells are bipotential and suggested to be the origin of breast cancer development, but are elusive and vaguely characterized. Breast tumors can be divided into subgroups, each one requiring specific treatment. To determine a possible association between mammary stem cells and breast cancer, a detailed characterization of the transcriptome in mammary stem cells is essential.

**Methods:**

We have used a murine mammary epithelial stem-like cell line (HC11) and made a thorough investigation of global gene-expression changes during stepwise differentiation using dual-color comparative microarray technique. Subsequently, we have performed a cross-species comparison to reveal conserved gene expression between stem cells and subtype-specific and prognosis gene signatures, and correlated gene expression to *in vivo *mammary gland development.

**Results:**

Our analysis of mammary stem-like and stepwise cell differentiation, and an in-depth description of our findings in a breast cancer perspective provide a unique map of the transcriptomic changes and a number of novel mammary stem cell markers. We correlate the alterations to *in vivo *mammary gland differentiation, and describe novel changes in nuclear receptor gene expression. Interestingly, our comparisons show that specific subtypes of breast cancers with poor prognosis and metastasizing capabilities show resemblance to stem-like gene expression.

**Conclusions:**

The transcriptional characterization of these mammary stem-like cells and their differentiation-induced gene expression patterns is here made widely accessible and provides a basis for research on mammary stem-like cells. Our comparisons suggest that some tumors are more stem-like than others, with a corresponding worse prognosis. This information would, if established, be important for treatment decisions. We also suggest several marker candidates valuable to investigate further.

## Introduction

The mammary gland exhibits unique developmental features during puberty, pregnancy and lactation. For each round of pregnancy, the mammary gland undergoes sequential cycles of proliferation, differentiation and apoptosis, and alveolar ducts form and grow, differentiate to produce milk and, after lactation, cease, revert and regress to the pre-pregnancy state. These and several other observations point towards the presence of stem cells as the basis for the capacity for alveolar renewal in each pregnancy (reviewed in [1]). These stem cells would be the origin of the epithelial compartment, where committed precursor cells become restricted to either a myoepithelial or luminal (ductal or alveolar) fate [1].

The cellular identity and markers of a rapidly cycling population of normal adult mammary stem cells have been suggested [2]. Some current models explain cancer development by the stem cell and clonal evolution hypothesis [3]. A bipotential mammary epithelial stem cell and/or a luminal progenitor cell would, via acquired genetic alterations, epigenetic changes and paracrine signals from surrounding cells, abandon its controlled stem cell self-renewal and develop into a breast tumor cell with uncontrolled growth [3,4]. Depending on the cell of origin (stem cell or progenitor cell) and acquired changes, different forms of breast cancer can develop. Tumors are further hypothesized to be sustained by the subpopulation of cancer stem cells [5], which should be specifically targeted for long-term successful therapeutic intervention.

The only factor known to consistently decrease lifetime breast cancer risk is early childbirth and breastfeeding [6]. This is speculated to be due to decreased circulating hormones, increased differentiation of the mammary cells and/or a decrease of mammary stem cells in the gland [7]. Exposure to estrogen is a risk factor for the development of breast cancer. The effects of estrogens are mediated via two estrogen receptor (ER) isoforms: ERα and ERβ. Activation of ERα drives the proliferation in most of ERα-positive breast tumors, whereas ERβ can behave as an antagonist to ERα at the transcriptional level [8] and may be of protective value in breast cancer. Epidemiological studies also suggest that the breast is at particular risk to acquire deleterious genetic changes before or during puberty, which is thought to be a period of stem cell expansion. At this time, exposure to phytoestrogens, ligands of ERβ, has also been shown to be protective [9].

The overwhelming majority of breast cancers have luminal epithelial characteristics. Five molecular subgroups of breast cancer have been defined that can be distinguished according to their gene expression profiles: basal like, luminal A, luminal C, Her2^+ ^and normal breast like [10]. Two nuclear receptors, ERα and its downstream target the progesterone receptor, are two of the most important prognostic markers of breast cancer, whose expression is an indication that anti-estrogenic therapy can be successful. Most luminal A and luminal C tumors express these markers, but luminal A tumors have a considerably better prognosis than luminal C tumors. Most basal-like, normal breast-like and Her2^+ ^subtypes do not express ERα; of these subtypes, Her2^+ ^and basal like have a worse prognosis [10]. There is a need for improved prognostic markers and tailor-made treatment for each of these groups.

The normal mammary epithelial cell line HC11 originates from mid-pregnancy BALB/c mice [11], and resembles mammary stem cells or progenitor cells remarkably. HC11 cells exhibit both self-renewal and ability for pluripotency: these cells can be cultured for an unlimited number of passages in a proliferating stem cell-like phase, they can be made to functionally differentiate and express milk proteins *in vitro *[11] and they can reconstitute the ductal epithelium of a cleared mammary fat pad with myoepithelial, alveolar and ductal luminal cells *in vivo *[12]. This cell line can be cultured without requirements for complex exogenously added, without extracellular matrix or without co-cultivation with other cell types for the prolactin-dependent *in vitro *induction of differentiation [11].

Few studies have been performed to investigate the underlying mechanisms when proliferating mammary epithelial cells differentiate using such pure cell systems of normal stem-like cells. Desrivières and colleagues have performed initial studies at the proteome level [13,14] in the HC11 cells, and different mammary epithelial subpopulations (basal/myoepithelial, luminal ERα-positive and luminal ERα-negative) were recently characterized at the transcriptome level [15]; however, a broad description of mammary stem cell or stem cell-like transcriptomes and their change during differentiation is lacking. *In vitro *models have the benefit of representing homogeneous cell populations, giving enough material for thorough, repeated and high-quality analysis, allowing manipulations to better understand development, characteristics and responses and minimizing animal use.

In the present study, the undifferentiated stage of the HC11 cells is referred to as stem like, since the cells have the capability of both self-renewal and pluripotency, but it is not entirely clear whether the HC11 cells are complete stem cells or are in the process of becoming progenitor cells. In order to investigate the transcriptional events behind this stem cell-like state, to provide a map of mammary epithelial cell differentiation and to determine a possible connection between mammary stem cells and breast cancer, we have performed a complete analysis of all 36,000 known genes and transcripts using dual-color microarray of the stem cell-like stage and different differentiation stages of these cells. We have compared this stem cell-like transcriptome with *in vivo *mammary gland development and with gene expression signatures of different types of human breast cancers and their metastases, and have analyzed the biological consequences in the context of stem cell and breast cancer interaction in detail. We report potential stem-like markers, and show their specificity at the protein level.

## Materials and methods

### Cell culture and induction of differentiation

HC11 mammary epithelial cells were routinely grown in complete medium (RPMI 1640, 10% FBS, L-glutamine, 5 μg/ml insulin, 10 ng/ml epidermal growth factor, and 50 μg/ml gentamicin; all from Sigma, St Louis, MO, USA), and proliferating cells were obtained under these growth conditions. When cells reached confluence, the complete medium was changed to medium without epidermal growth factor (RPMI 1640, 2% FBS, 5 μg/ml insulin, and 50 μg/ml gentamicin), and pre-differentiated (competent) cells were obtained after 48 hours of growth in this medium. To induce differentiation of competent cells, cultures were treated for 72 hours with medium without epidermal growth factor containing 100 nM dexamethasone and 1 μg/ml ovine prolactin.

### Animals and mammary gland tissue

Female Balb/c mice were fed *ad libitum *and were kept under a 12-hour light, 12-hour dark cycle. Mammary glands from 2-month old virgin, 10-day pregnant and 6-day lactation mice were excised and frozen in liquid nitrogen for RNA extraction. All animal experimentation was conducted in accordance with accepted standards of humane animal care as outlined by the Stockholm South Ethical Committee of the Swedish National Board for Laboratory Animals.

### RNA extraction

RNA was extracted with TRIzol (Invitrogen, Carlsbad, CA, USA) according to standard protocol, and purified using Qiagen RNeasy spin columns (Qiagen, Hilden, Germany) with on-column DNase I digestion.

### Microarray experiment and analysis

Raw data and detailed protocols for the microarray analysis are available from the ArrayExpress data repository [E-MEXP-969]. In short, Operon's long-oligonucleotide spotted arrays covering the whole known genome represented by 36,000 genes and gene transcripts were used (printed by the microarray facility at KTH, Royal Institute of Technology, Stockholm, Sweden). Triplicate cultures of stem-like, pre-differentiated and differentiated stages of HC11 were analyzed in 10 hybridizations using 20 μg total RNA per sample (20 samples); each microarray comparison was replicated and dye-swapped with different sets of cultures to compensate for unequal dye incorporation and variance between cultures and differentiations. Hybridizations were performed in a loop design, where stem-like stage was hybridized to pre-differentiated stage, pre-differentiated stage to fully differentiated stage and, in addition, stem-like stage to fully differentiated stage. Scanning was carried out at 10 μm resolution using the G2565BA DNA microarray scanner (Agilent Technologies, Santa Clara, CA, USA), for which the photomultiplier tube was set to 100.

The obtained TIFF images were analyzed using the GenePix Pro 6 software (Molecular Devices Corp., Union City, CA, USA), in which TIFF files generated for Cy3 channels and Cy5 channels were superimposed upon each other. Spot identification, manual examination of the surface of the array and flagging of spots/regions with poor quality were performed in GenePix. Result files (gpr files) produced by GenePix were imported into the R environment for statistical computing and programming, where data processing and identification of differentially expressed genes were carried out using the Bioconductor package bundle, Limma, aroma package and the kth-package. Unreliable spots with abnormal physical properties were removed using several filters. After filtering, the slides were normalized with print-tip loess (local regression) normalization. To identify differentially expressed genes, a parametric empirical Bayes approach implemented in Limma was used [16]. This test statistic will assign a score (B-score) to each gene. The B-score was used to rank the genes so that the gene with the highest score has the highest probability of being differentially expressed. When differences were being investigated, two criteria had to be fulfilled for a gene to be regarded as differentially expressed: the gene had to have a B-score of more than 0 and an |M-value| of more than 0.5 (an M-value is the second logarithm of the fold change).

Genes close to cut-off (B > 0.0, |2logFC| > 0.4 and *P *< 0.005) could be confirmed by real-time PCR in three independent differentiation cultures. Confirmations of 22 genes were performed.

### Over-representation analysis

Analysis of over-represented themes and classification into gene ontology functional groups was performed using Expression Analysis Systematic Explorer [17]. The complete mouse transcriptome was used to calculate expected frequencies of over-represented themes. Gene ontology groups were considered over-represented when the calculated Expression Analysis Systematic Explorer score (modified Fischer Exact probability test) was ≤ 0.2.

### Real-time PCR

cDNA was synthesized with Superscript III (Invitrogen) and random hexamers using 1 μg DNase-I-treated and purified RNA. Real-time PCR was performed with SYBR-Green Mastermix (Applied Biosystems) according to the manufacturer's protocol, using 10 ng cDNA per 10 μl reaction in an ABI PRISM 7500 (Applied Biosystems, Foster City, CA, USA) under the following conditions: 95°C for 10 minutes, followed by 40 cycles at 95°C for 15 seconds and 60°C for 40 seconds. All runs were performed in triplicate from triplicate cultures, and specific amplification was checked with melting curve analysis. Primer sequences of intron-spanning fragments will be provided on request. The ΔCt formula was used to determine fold-change differences, and 18S was used as the reference gene.

### Western blot

HC11 cells were grown in 10 cm plates and were differentiated as described above. Cells were washed with PBS, collected in a 1.5 ml tube, and pelleted by centrifugation at 4°C for 2 minutes. The cell pellets underwent one cycle of freeze–thaw and were resuspended in lysis buffer (1% NP-40, 50 mM Tris–HCl, pH 7.5, 140 mM NaCl, 2 mM ethylenediamine tetraacetic acid, 1 mM phenylmethanesulphonylfluoride, 1 mM Na_3_VO_4 _and protease inhibitor cocktail; Roche, Mannheim, Germany). The lysate was kept on ice for 20 minutes and was centrifuged at 14,000 rpm for 10 minutes at 4°C. The protein concentration was determined with Bradford reagent (BioRad, Hercules, CA, USA). Whole-cell extracts (40 μg protein) were resolved on SDS-PAGE and transferred onto a Polyvinylidene Fluoride membrane.

The membranes were blocked with 5% (w/v) milk protein dissolved in PBS and were incubated overnight at 4°C with the following primary antibodies: rabbit anti-ADAMTS1 (GTX11557; Genetex, Irvine, CA, USA), chicken anti-DUSP6 (GTX19115; Genetex), rabbit anti-Birc5 (also known as survivin, #2808; Cell Signaling, Houston, TX, USA), rabbit anti-Melk (#2274; Cell Signaling), and mouse anti-COUP TF2 (ab41859; AbCam, Cambridge, UK) and rabbit anti-tubulin (#2125; Cell Signaling) was used as loading control. The antibodies were considered specific based on previous data and detection of few bands in the blots, and in all cases the molecular weight of the corresponding bands was calculated using Quantity One software (BioRad). The secondary antibodies were coupled to horseradish peroxidase (Sigma). The luminescent signal was detected with the enhanced chemiluminescence kit (Amersham, Buckinghamshire, UK).

### Immunocytochemistry and immunohistochemistry

HC11 cells were cultured on eight-well glass chamber slides (BD Biosciences, Meylan Cedex, France). Cells were fixed in 10% buffered formalin and permeabilized in 0.5% Triton–PBS for 30 minutes. Unspecific binding was blocked by incubation in blocking solution for 1 hour (10% FBS in 0.1% Tween–PBS). Incubation with the primary antibodies proceeded overnight at room temperature. The primary antibodies used were the same as for western blots and also anti-mouse CD44 (#553991; BD Pharmigen, San Diego, CA, USA), mouse anti-keratin 5/8 (MA1-35858; Affinity Bioreagents, Rockford, IL, USA) and mouse anti-Rab4 (ab13252; AbCam). After three washes with PBS, the corresponding secondary antibodies – anti-mouse-Cy3 (Sigma), anti-rabbit or anti-chicken Alexa 488 (Molecular Probes, Invitrogen, Eugene, OR, USA) and anti-mouse Alexa 568 (Molecular Probes) – were added (1/500 in blocking solution) for 1 hour. Cells were washed four times with PBS and nuclei were stained with ToPro-3 (1/1,000; Molecular Probes). To assess whether the staining was due to binding of the primary antibody, a group of samples was stained in parallel but only with the secondary antibody. Images were captured using a laser-scanning confocal microscope (Zeiss 510, Stockholm, Sweden). Images were acquired under the same settings; when edited with Adobe Photoshop 6.0, the same adjustments were applied to all images.

## Results and discussion

### Mammary stem-like cell transcriptome

**Figure 1:**
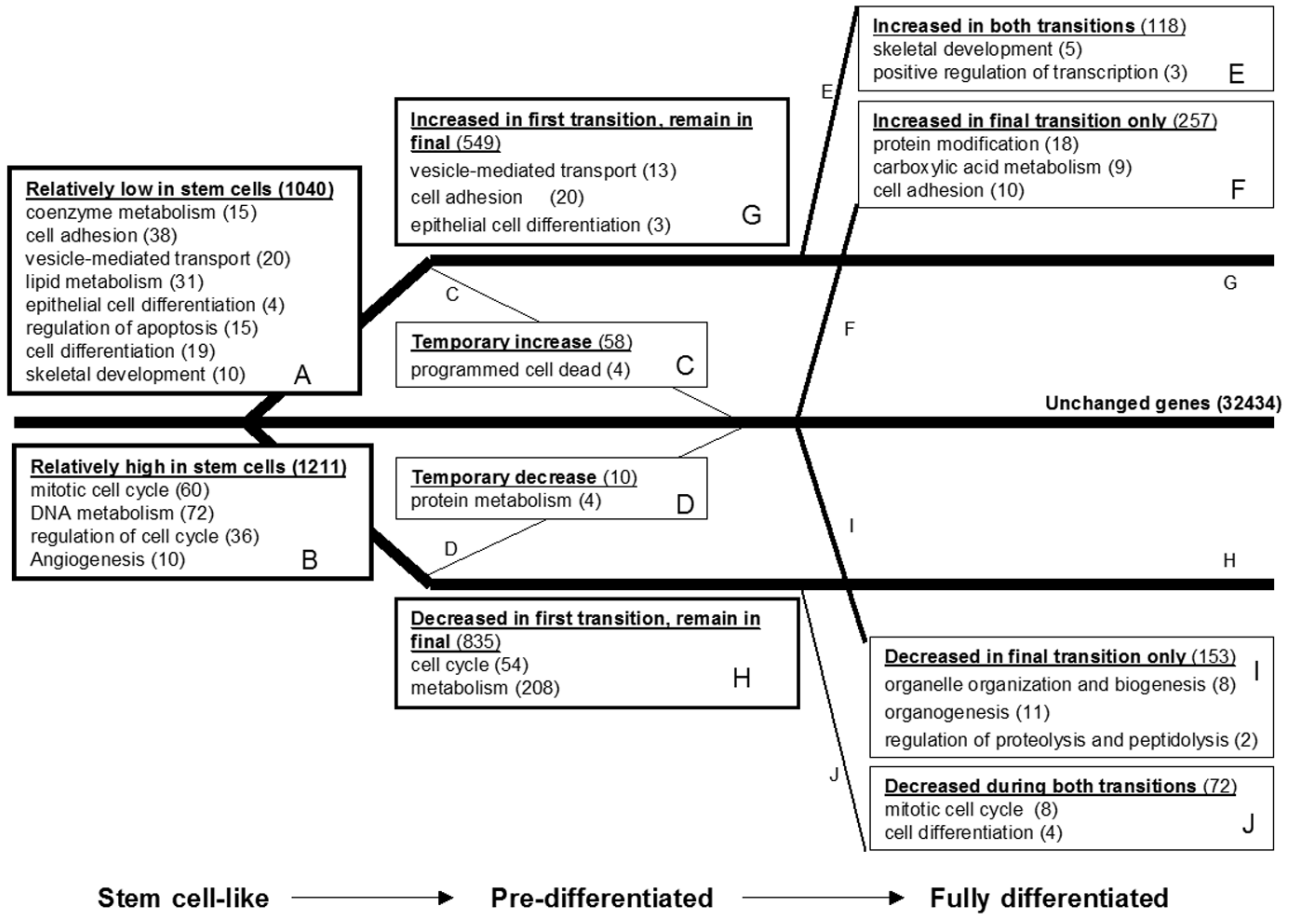
Schematic overview of mammary stem cell-like cells to differentiation transcriptome transition. Gene categories (as defined by the Gene Ontology Consortium) over-represented among differentially expressed genes during the transition from stem-like mammary epithelial cells via pre-differentiated cells to fully differentiated cells. Over-representation analysis was performed using Expression Analysis Systematic Explorer (EASE) software, with an EASE score (a conservative adjustment of Fisher's exact probability to weigh significance in favor of gene ontology categories supported by many genes) cut-off below 0.02. Boxes A and B denote overall changes between the first and second transitions (stem cell-like stage compared with pre-differentiated stage). Boxes C to J denote subgroups where a changed or unchanged value is acquired in the microarray analysis for both transitions (stem cell-like stage compared with pre-differentiated stage, as well as pre-differentiated stage compared with fully differentiated stage). Number of genes within each group and process are shown within parentheses.

**Figure 2:**
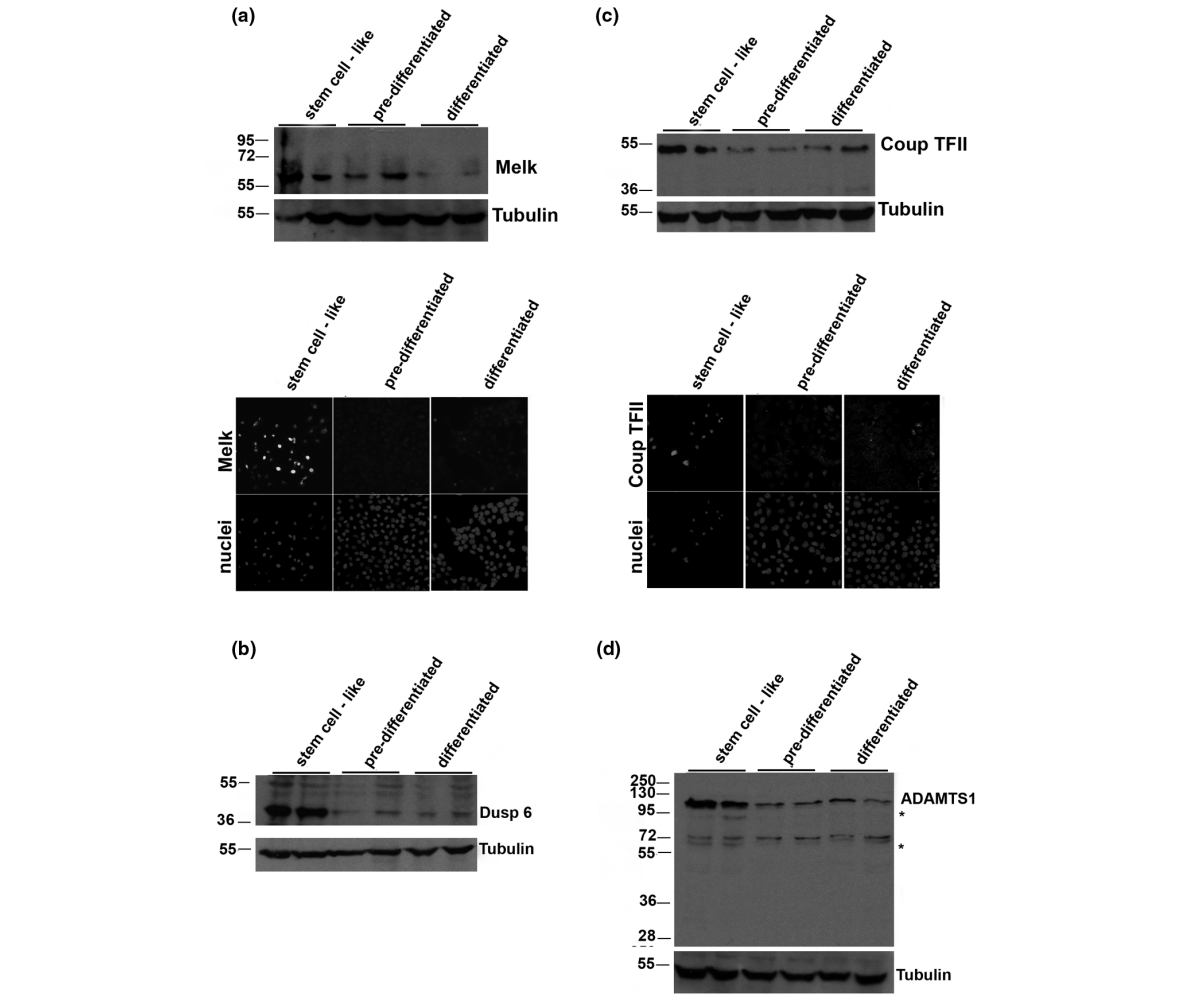
Protein expression in HC11 stem-like, pre-differentiated and differentiated cells. **(a) **Melk expression analyzed by western blot and immunofluorescence. **(b) **Dusp 6 expression by western blot. **(c) **COUPTF-II expression by western blot and immunofluorescence. **(d) **ADAMTS1 expression analyzed by western blot. *Processed forms of the zymogen. In all cases, whole cell extracts were resolved on 10% SDS-PAGE and membranes blotted with the indicated antibodies. Tubulin was used as loading control.

**Figure 3:**
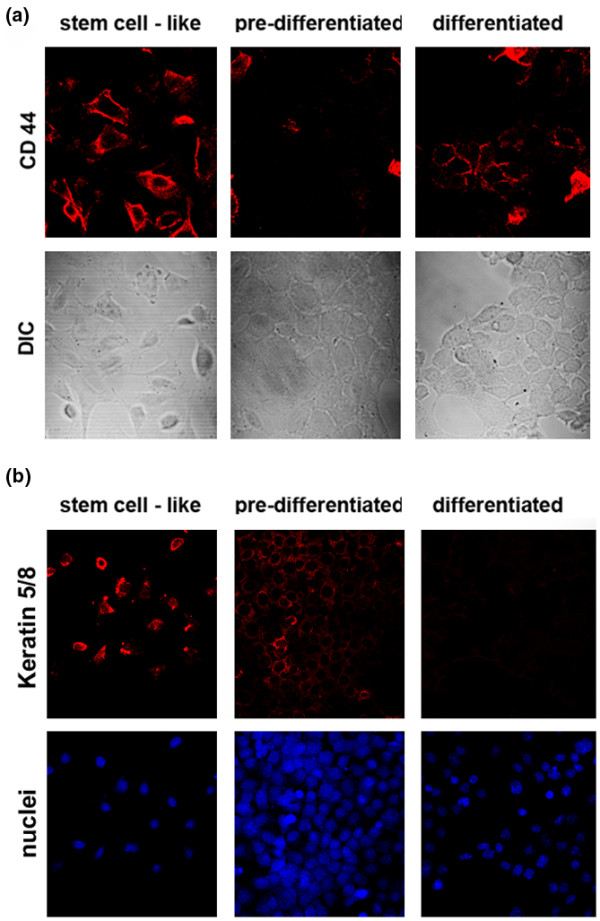
Expression of stem cell markers in HC11 mammary epithelial cells. **(a) **CD44 expression. DIC, differential interference contrast allows visualization of the cells. Note that the proportion of CD44-positive cells decreases as the culture differentiates but the intensity of the signal in those cells that are positive remains constant. **(b) **Keratin 5 and Keratin 8 (Krt 5/8) expression.

**Figure 4:**
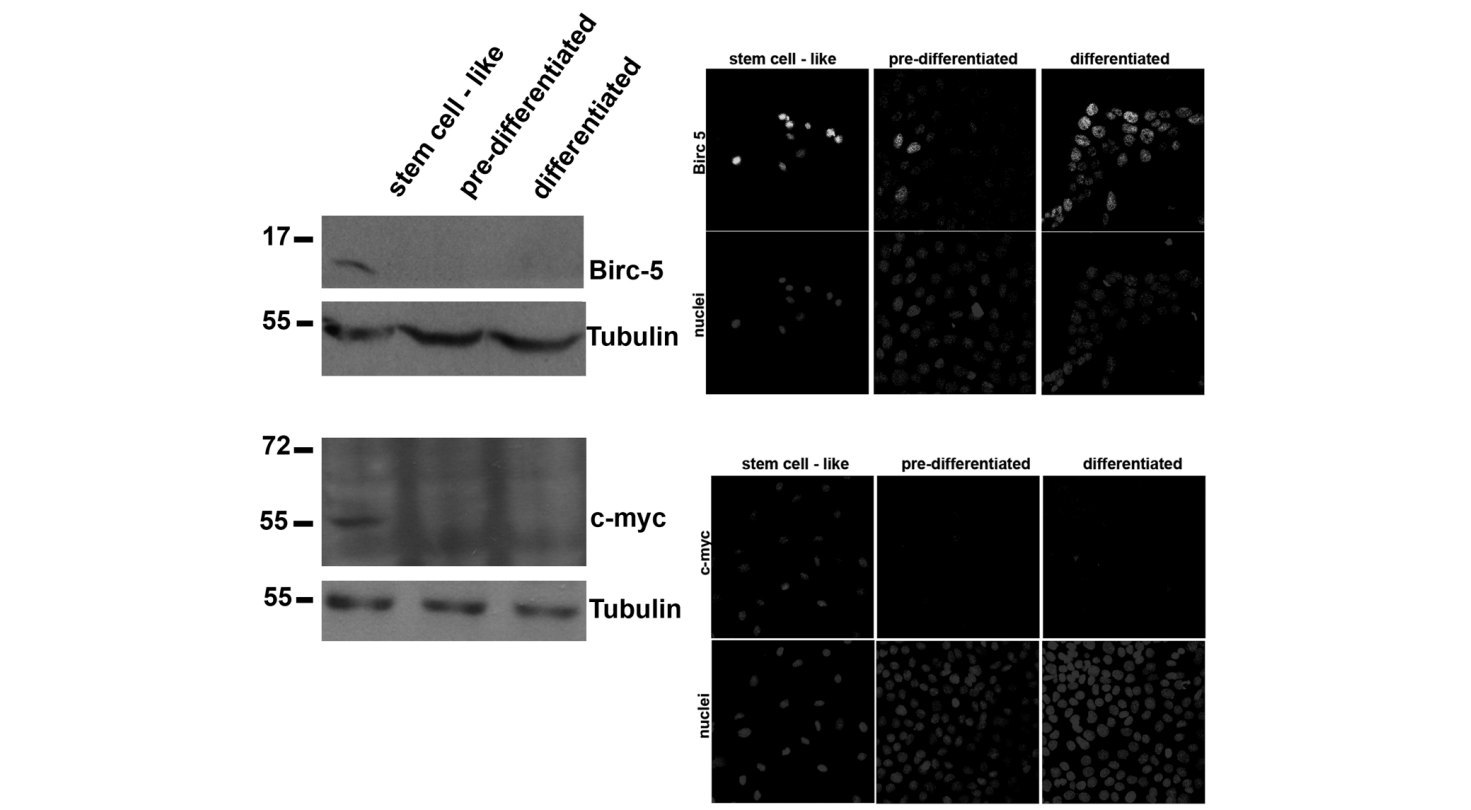
Expression of Wnt regulated genes in HC11 stem cell-like, pre-differentiated and differentiated cells. Wnt regulated gene expression evaluated by western blot and immunofluorescence in HC11 stem cell-like cells, pre-differentiated cells and differentiated cells. Top panel, Birc5; bottom panel, c-myc. In all cases, whole cell extracts were resolved on 12% SDS-PAGE and membranes were blotted with the indicated antibodies. Tubulin was used as loading control.

**Figure 5:**
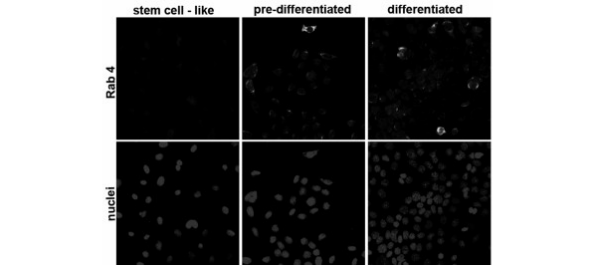
Rab4 expression in HC11 stem-like, pre-differentiated and differentiated cells. Rab4 expression analyzed by immunofluorescence in HC11 stem-like cells, pre-differentiated cells and differentiated cells.

To explore the largely unknown transcriptome of mammary stem cells we compared the murine mammary stem-like cells with their pre-differentiated and fully differentiated stage. In this way, all changes of genes and important networks for commitment of cell fate, implementation of differentiation programs, specification and survival were revealed. The most highly changed genes, at stem cell-like exit, are presented in Tables 1 and 2, gene enrichment analysis of over-represented and under-represented processes is listed in Table 3, and a schematic overview of changed processes is depicted in Figure 1. All regulated genes are available in Additional file 1, and real-time confirmations of 22 genes are available as Additional files 2 to 4. Confirmations at the protein level are depicted in Figures 2 to 5.

**Table 1:** Mammary stem-like specific gene expression

Symbol	Gene	2logFC	Gene ontology
Hist1h2bp	Histone 1, H2bp	-2.78	Nucleosome assembly
Hist1h2bk	Histone 1, H2bj	-2.95	Nucleosome assembly
Hmmr	Hyaluronan-mediated motility receptor	-2.85	Not defined
Melk	Maternal embryonic leucine zipper kinase	-2.68	Protein amino acid phosphorylation
Cbln4	Cerebellin 4 precursor protein	-2.38	Not defined
Hist2h2ac	Histone 2, H2ac	-2.55	Nucleosome assembly
Ccna2	Cyclin A2	-3.21	Regulation of cell cycle
Hist2h3c1	Histone 2, H3c1	-2.91	Nucleosome assembly
Birc5	Baculoviral IAP repeat-containing 5	-2.55	Anti-apoptosis/embryonic development
Exosc6	Exosome component 6	-2.10	rRNA processing
Tmpo	Thymopoietin	-1.60	Regulation of transcription
Hist1h4h	Histone 1, H4h	-1.99	Nucleosome assembly
Lig1	Ligase I, DNA, ATP-dependent	-1.97	Cell cycle
Dusp6	Dual-specificity phosphatase 6	-2.80	Protein amino acid dephosphorylation
Hist1h3c	Histone1, H3c	-2.62	Nucleosome assembly
Ckap2	Cytoskeleton-associated protein 2	-1.97	Cell cycle/apoptosis
Adamts1	A disintegrin-like and metalloprotease with thrombospondin type 1 motif, 1	-2.47	Integrin-mediated signaling pathway/proteolysis
Hist1h4a	Histone 1, H4a	-2.23	Nucleosome assembly
Mki67	Antigen identified by monoclonal antibody Ki67	-2.00	Cell proliferation
H2afz	H2A histone family, member Z	-2.53	Nucleosome assembly/multicellular organismal development
Cyr61	Cysteine-rich protein 61	-2.89	Regulation of cell growth/cell adhesion
Rrm1	Ribonucleotide reductase M1	-2.44	DNA replication
Hist1h2ag	Histone 1, H2ag	-2.96	Nucleosome assembly
Lyar	Ly1 antibody reactive clone	-1.81	Not defined
Ube2c	Ubiquitin-conjugating enzyme E2C	-1.89	Positive regulation of cell proliferation

Genes highly expressed at the stem-like stage, to decrease upon differentiation. Genes are listed in the order of statistical significance for being differentially expressed. 2logFC denotes the 2log of fold-change (a value of -2 equals a fourfold downregulation or a decrease by 75% upon differentiation).

**Table 2:** Mammary differentiation specific gene expression

Symbol	Gene	2logFC	Gene ontology
Expi	Extracellular proteinase inhibitor	5.01	Protease inhibitor activity
Gas6	Growth arrest specific 6	3.14	Regulation of cell growth
Slc6a6	Solute carrier family 6, member 6	2.88	Neurotransmitter transport
Ehf	Ets homologous factor	3.66	Transcription factor activity
Ctsd	Cathepsin D	2.88	Proteolysis
Xdh	Xanthine dehydrogenase	1.83	Lactation/regulation of epithelial cell differentiation
D0H4S114	Dna segment, human D4S114	3.32	Regulation of Tgf-b signaling pathway
Nupr1	Nuclear protein 1	1.92	Not defined
H2-T23	Histocompatibility 2, T region locus 23	1.75	Antigen processing and presentation
Atp6v0d1	ATPase, H transporting, V_0 _subunit D isoform 1	1.78	Proton transport
Cd81	CD 81 antigen	2.03	Positive regulation of cell growth
D12Ertd647e	DNA segment, Chr 12, ERATO Doi 647, expressed, transcript variant 3	2.22	Not defined
Cbr2	Carbonyl reductase 2	2.92	Metabolic process
Plagl1	Pleiomorphic adenoma gene-like 1	2.43	Positive regulation of transcription from RNA polymerase II promoter
Atp6v1a1	ATPase, H transporting, V_1 _subunit A, isoform 1	1.96	Proton transport
Ctsa	Cathepsin A	2.17	Proteolysis
Stat1	Signal transducer and activator of transcription 1	1.56	Transcription
Oas1a	2',5' -Oligoadenylate synthetase 1A	1.61	Negative regulation of viral reproduction
Ddx58	DEAD box polypeptide 58	1.90	Immune response
Tmem154	Transmembrane protein 154	1.76	Not defined
Fcgrt	Fc receptor, IgG, alpha chain transporter	1.41	Immune response
Itm2b	Integral membrane protein 2B	2.45	Induction of apoptosis
Sema6a	Sema domain, transmembrane domain and cytoplasmic domain (semaphorin) 6A	1.52	Cell differentiation/apoptosis
Cuedc1	CUE domain containing 1	1.54	Not defined
Rtp4	Receptor transporter protein 4	1.96	Not defined

Genes that increase strongly upon initiation of differentiation. 2logFC denotes the 2log of fold-change (a value of 2 equals a fourfold upregulation or a 400% increase during differentiation).

**Table 3:** Biological functions over-represented and under-represented in HC11 mammary stem-like cells

	No genes	EASE score
Over-represented in stem-like cells		
Cell cycle	90	3.6 × 10^-27^
DNA metabolism	72	1.7 × 10^-23^
RNA metabolism	45	3.2 × 10^-12^
Regulation of cell cycle	36	1.8 × 10^-7^
Chromatin assembly/disassembly	14	2.0 × 10^-5^
Protein amino acid phosphorylation	34	0.008
Protein metabolism	118	0.01
Under-represented in stem-like cells		
Coenzyme metabolism	15	0.0003
Cell adhesion	38	0.0007
Vesicle-mediated transport	20	0.003
Lipid metabolism	31	0.004
Epithelial cell differentiation	4	0.005
Regulation of apoptosis	15	0.005
Cell differentiation	19	0.03
Skeletal development	10	0.05
Regulation of transcription, DNA dependent	72	0.13

Biological function as defined by gene ontology. The stem-like cell gene expression is compared with expression in the pre-differentiated stage. Expression Analysis Systematic Explorer (EASE) score: modified Fischer exact probability *t *test.

Our analysis confirmed previous findings, including: an increase of Stat1 and Stat5a during differentiation, which also mimics the *in vivo *control of proliferation, differentiation and survival during mammary gland differentiation [18]; an increase of Ctgf expressed, also found in the *in vivo *mouse mammary gland during pregnancy and lactation [19]; an increase of Lamp1, a differentiation marker for HC11 [20]; and an increase of Igfbp5, whose expression is stimulated during cellular differentiation by lactogenic hormones [21]. Further, a decrease of Igfbp2 [21], Hnrpd [22], Cyclin D_1 _and Myc [23] during the differentiation of these cells was confirmed. In addition to the above verifications, which in themselves demonstrate a high reproducibility of the differentiation process and of the analysis, a total of 2,251 genes was observed to be changed in the first step of commitment to differentiation, and a further 1,010 alterations during the subsequent stage of functional differentiation. The majority of these alterations are novel, and the implications of many of them are at this time unknown.

When cells left the stem-like stage and entered the pre-differentiation stage, gene enrichment analysis of processes – as defined by the Gene Ontology Consortium – showed that differentiation (particularly epithelial differentiation), skeletal development, cell adhesion, regulation of apoptosis and several types of metabolisms (coenzyme, lipid, carbohydrate, oxygen and sulfur metabolism) increased (Figure 1, box A). At the same time, there was a robust decrease in expression of mitotic cell cycle-associated genes, metabolism of DNA, RNA, nucleotide and proteins, respectively, and angiogenesis (Figure 1, box B). Most genes that were altered in the first transition remained at this level in the second transition, such as upregulated genes within vesicle-mediated transport, cell adhesion and epithelial cell differentiation (Figure 1, box G). Expression of other genes continued to increase: for example, skeletal development and positive regulation of transcription (Figure 1, box E). There was also a significant increase of protein modifications and additional cell adhesion gene expression specific for the second transition (Figure 1, box F). The strong slow-down of proliferation in the first differentiation transition partially continued in the second differentiation step (Figure 1, box J – also signified by a strong decrease in proliferation marker Mki67), whereas most of the decreased cell cycle genes did not change further in the second transition (Figure 1, box H). Organelle organization, biogenesis, organogenesis and regulation of proteolysis and peptidolysis started to decrease in the second transition (Figure 1, box I). A temporary increase of 58 genes, pronounced within programmed cell death genes (Figure 1, box C), and a temporary decrease of protein metabolism genes (Figure 1, box D) were specific changes that only occurred in the pre-differentiated stage.

Among the most strongly altered genes (Tables 1 and 2), transcript levels of amino acid phosphorylation proteins (including Melk) and dephosphorylation proteins (including Dusp6) were highly elevated at the stem-like cell stage and decreased considerably during differentiation. Melk and Dusp6 expression was further corroborated at the protein level (Figure 2a, b), where the decrease upon differentiation was evident. A large group of different histone transcripts were also found to be strongly downregulated during differentiation – for example, Hist1h1a confirmed by real-time PCR (Additional file 2), Hist1h4h, Hist1h3c and H2afz (Table 1). When differentiation starts, gene expression of proteinase inhibitor activity (Expi, confirmed by real-time PCR; Additional file 2), proteolysis proteins (such as cathepsin D and cathepsin A) and transcription-related proteins (Ehf, confirmed by real-time PCR (Additional file 2), Plagl1, Stat1, Stat3, Stat5a and Stat6) increased. From these data we can conclude that the HC11 stem-like cells have a high activity of cell cycle, protein phosphorylation and angiogenic activities coupled with low adhesion, apoptosis, transcriptional activity and differentiation.

### Mammary stem cell characteristics in the HC11 stem-like cells

The exact features of mammary stem cells are not fully known, and there are disagreeing reports of mammary stem cell characteristics. Also the exact stem cell characteristics of the cell line used here are unspecified, but exhibit several trademarks of stem cells. The HC11 cells are immortal and have the capability of self-renewal and pluripotency, and maintain properties that allow them to differentiate *in vitro *in response to lactogenic hormones. Their repopulation potential *in vivo *may not be perfectly reproducible, however, and the expression of stem cell markers is not well established. Further, these cells are proliferating, which our current dogma suggests stem cells of the mammary gland are not. These cells may be in the process of becoming progenitor cells, or they may possibly constitute a mixture of stem and progenitor cells. Regardless, it is of great interest to define the potential stem-like gene expression pattern and/or markers for these cells.

To evaluate our material for stem cell characteristics, we compared the gene expression of the stem-like stage with findings reported or suggested by others. A bipotential human stem cell is hypothesized to be Cd44^+ ^[3], an adhesion molecule with roles in signaling, migration and homing. We found that Cd44 was highly expressed at the stem-like cell stage and decreased extensively during differentiation at the mRNA level. Cd44 at the protein level also decreased with differentiation (Figure 3a), although occasional cells remained positive also in the differentiated stage. Furthermore, a receptor with similar properties to Cd44, Hmmr, was strongly overexpressed in the stem-like stage. A strong correlation with other suggested mammary stem cell marker genes was observed; that is, Brca1 [24], Krt6, Krt5 [25] and Melk, a suggested stem cell gene in hematopoietic [26], neural [27] and possibly epidermal stem cells – the latter two genes were observed and corroborated also at protein level (Figure 3b and Figure 2a, respectively).

A novel mechanism for the control of stem cell proliferation in embryonic and neural stem cells involving histone H2afx was recently described [28]. We found this gene to be overexpressed in our stem-like cells. This histone gene has also been shown to exhibit copy number changes in sporadic breast cancer [29]. Vim (vimentin), a suggested stem cell marker in mesenchymal cells [30], also showed a decrease during differentiation (corroborated at protein level; Additional file 6). Other possible stem cell markers, however, showed an opposite expression in these cells: Lrp5 (a reported cell surface marker for somatic mammary stem cells), Musashi homolog 2 (Msi2, a neuronal stem cell marker) and both Kit and Kitl (stem cell markers in hematopoietic stem cells) were all upregulated at the transcript level during differentiation of HC11 mammary stem cell-like cells.

Murine mammary stem cells have further been selected using protein markers CD45/Ptprc^-^, Ter119/Ly76^-^, CD31/Pecam1^-^, Sca-1/Ly6a^low^, CD24/Cd24a^med ^and CD49f/Itga6^high ^by Stingl and coworkers [2]. In our HC11 material, however, these markers were not significantly changed at the transcript level. One reason for these apparent differences may be that the protein levels at the cell surface do not always follow the mRNA levels, so even if the transcript for a specific gene does not change, other mechanisms can affect both protein localization and stability. Another reason could be related to where in the process between pure stem cells towards progenitor cells our stem-like cells and/or the literature reported cells are residing. The transcription factor Etv4 (Pea3), suggested to function in multipotential mammary progenitors to regulate their lineage-specific differentiation potential by Kurpios and colleagues [31], was at the highest expression in the stem-like stage.

HC11 cells have the capacity to differentiate *in vivo *into both myoepithelial and luminal (ductal and alveolar) epithelial cells. Both markers of myoepithelial lineage (Mme/Cd10) and luminal epithelial lineage (Krt18) increased strongly during differentiation. Further, we compared the expression patterns of the HC11 cell differentiation stages with the three different mammary epithelial cell subpopulations – basal/myoepithelial, luminal ERα-positive and luminal ERα-negative – from virgin mouse mammary gland, characterized at the transcriptome level in a study by Kendrick and colleagues [15]. Genes specific for each of the three subgroups were increasing in expression during differentiation of the HC11 cells, further proving that all lineages are represented in the differentiated stage. We found that whereas nearly all luminal specific genes that were changed during the differentiation of HC11 cells were upregulated (79 out of 82 for luminal ER-positive and 79 out of 84 for luminal ER-negative), about one-half of the genes specific for basal/myoepithelial lineage (99 genes) were upregulated during differentiation, whereas the remaining 91 genes decreased. The reason for this could be that, because the basal cell layer also contains the mammary epithelial stem cell compartment [32], and genes in the stem cells should be downregulated during differentiation, genes in differentiated cells in the basal/myoepithelial lineage should be correspondingly upregulated during differentiation. The luminal cells should be mostly represented by differentiated cells, and genes in these cells would be expected to be upregulated during HC11 differentiation.

During midgestational mammary development *in vivo*, several signal transducers and activators of transcription are known to be increased by prolactin; in the HC11 cells, Stat1 (Table 2), Stat3, Stat5a and Stat6 as well as the prolactin-induced protein increased, indicating that the cells retain this mammary epithelial cell-differentiation attribute. Furthermore, the milk protein β-casein was expressed in differentiated cells (Additional file 7).

Activity of the Wnt, Hedgehog and Notch pathways are other hallmarks of stem cell characteristics. In the HC11 cells, drastic changes of expression of Wnt members during differentiation was not observed; however, several known positively regulated Wnt target genes [33] were strongly downregulated during differentiation (Ccnd1, Birc5, Cd44 and Myc, all corroborated at mRNA and/or protein level (Figures 3 and 4, Additional file 2), and Cyr61, Fosl1, Cd87/Plaur, Met, Fst, Emp1, Abcb1b, Ptgs2, Abcb1b, Runx2, Gja1, IL6, Mycbp), and targets known to be downregulated by Wnt were correspondingly upregulated (Cdh1, confirmed at mRNA level (Additional file 2), Sox9 and Postn), in agreement with a higher Wnt activity at the stem-like cell stage. This normal downregulation of the Wnt pathway during differentiation correlates to a previous report by Shackleton and colleagues showing that normal differentiation could be inhibited by the over activation of the Wnt pathway [32]. Several members of the Hedgehog pathway (including Snail and Prkca) and the Notch pathway (including Jag1, Jag2, Hr, Lfng, Hes1) were also changed during differentiation.

A large study of heterogeneous collections of gene expression data generated from 83 mouse stem cell-related samples defined four super-families of stem cell markers associated with differentiation: serine proteinase inhibitors (serpins), cytochrome P450 family, Rab family GTPases, and nuclear receptors [30]. In HC11 mammary cells, differentially expressed genes signified all of these four groups. Two serpins (Serpine1 and Serpine2) were highly reduced as the stem-like cells underwent differentiation. Serpine1 is also involved in regulation of angiogenesis in breast cancer, and Serpine2 in cell differentiation. In a previous study of differentiating hematopoietic stem cells we also identified two serpins as strongly reduced (Serpin a3g and a3n) [34], of which expression of Serpina3g has been shown to prevent stem cells from differentiation [35]. We further found an increase of Group 2 genes (two cytochrome P450 members, Cyp2f2 and Cyp4x1, in the HC11 cells) and several Rab family GTPases in Group 3 (increase of Rab4a (confirmed at protein level in Figure 5), Rab1, Rab3a, Rab5b, Rab15, Rab18, Rab25, and related gene family members Rhoj, Rhoq, Rhou; and decrease of two Rab family GTPases Rab12 and Rab32) as the mammary stem-like cells differentiated.

Group 4 (nuclear receptors) was also differentially expressed in HC11 mammary stem-like cells. Ten nuclear receptors changed their expression when the mammary stem-like cells differentiated. COUP-TFII, COUP-TFI, FXRβ, NGFIB, NURR1 and ERβ decreased (Additional file 5). COUP-TFII and COUP-TFI influence proliferation of breast cancer cells [36,37] and are implicated in metastasis [38]. In the fly the common ancestral gene (svp/NR2F3) regulates stem cell identity of neuroblasts [39]. Figure 2c clearly shows the downregulation of COUP-TFII also at the protein level when the cells start differentiating. Further, RORα, VDR, EAR2 and ERα increased (Additional file 4). RORα is frequently inactivated in breast cancers, and VDR is indicated to be protective against breast cancer [40]. Both COUP-TFII and RORα are among 426 selected markers of stem cells described [30]. Further, we noted that both ERα and ERβ were among the nuclear receptors that changed during differentiation. Both are involved, in opposing manners, in mammary development and breast cancer [8].

Taken together, a number of stem cell-related changes as well as changes indicative of a mixture of both myoepithelial and luminal cell fates are in line with the stem cell characteristics of these cells.

### Mammary stem-like cells show resemblance with breast cancer signatures

To investigate whether there are similarities between human breast cancer gene expression and the stem cell-like expression of the murine HC11 cells, supporting the hypothesis that breast cancer primarily arises from mammary stem/progenitor cells [41], we compared our material of mammary stem-like gene expression with gene-profiling signatures of breast tumors. These profiles have a prognostic value equal to or better than clinicopathologic variables [42], and a 70-gene signature is able to distinguish sporadic breast cancer tumors with poor prognosis from those with favorable prognosis [43]. Comparison showed that the poor prognosis signature overlapped with the expression profile of the mammary stem cell-like genes. In contrast, none of the genes indicating favorable prognosis were differentially expressed in the stem-like cells (Table 4).

**Table 4:** Correlation of mouse mammary stem-like gene expression and breast tumor prognosis signatures

High at stem-like stage	Low at stem-like stage	Unchanged
**Poor prognosis**: 37 genes, 22 of which (59%) changed in stem-like stage		
17 genes	5 genes	15 genes

Melk – protein phosphorylation	Gpr126 – neuropeptide signaling pathway	Tmeff1 – development
Diap3 – cytoskeleton organization	Akap2 – unknown function	Exoc7 – protein transport
Ext1 – ossification	Oxct1 – metabolic process	Slc2a3 – transmembrane transport
Ect2 – signaling cascade	Fbxo31 – ubiquitin-dependent protein catabolic process	Lpcat1 – metabolic process
Uchl5 – ubiquitin-dependent protein catabolic process	Igfbp5 – regulation of cell growth	Egln1 – oxygen homeostasis
DC13 – unknown function	Esm1 – regulation of cell growth	Pitrm1 – proteolysis
Gmps – purine base biosynthetic process		Cdc42bpa – protein phosphorylation
Dck – pyrimidine nucleotide metabolic process		Gpr180 – unknown function
Rcf4 – DNA replication		Mmp9 – regulation of apoptosis
Orc6l – DNA replication		Hrasls – regulation of cell growth
Dtl – DNA replication		Flt1 – regulation of cell proliferation
Cenpa – nucleosome assembly		
Prc1 – cell cycle		
Ccne2 – cell cycle		
Kntc2 – cell cycle		
Mcm6 – cell cycle		
Nusap1 – cell cycle		

**Good prognosis**: 12 genes, none of which (0%) changed in stem-like stage		
0 genes	0 genes	12 genes

		Ap2b1 – protein transport
		Ms4a7 – signal transduction
		Stk32b – protein phosphorylation
		Scube2 – calcium ion binding
		Aldh4a1 – proline catabolic process
		Gstm3 – metabolic process
		Peci – metabolic process
		Ebf4 – regulation of transcription
		Bbc3 – induction of apoptosis
		Tgfb3 – cell growth/signal transduction
		Fgf18 – regulation of cell proliferation
		Wisp1 – regulation of cell growth

From van't Veer *et al*. [43]. Names and biological process (as defined by gene ontology, selected and/or abbreviated to fit table) are provided.

Among signatures distinguishing subclasses of breast carcinomas [10], our stem-like signature showed an irrelevant overlap with subclasses nominated as normal breast like, as luminal epithelial containing ER, and as Her2^+^. For subtype luminal C and basal-like tumors, however, there was a considerable overlap of genes (75% and 60%, respectively; see Table 5). Patients with these two subclasses of breast tumors also show among the lowest survival [10]. The basal-like phenotype has been suggested to resemble normal mammary stem cells [1,44], which is here demonstrated at the gene expression level. Both the HC11 cells and the poor-prognosis and basal-like tumors are characterized by high proliferation; nonetheless, many of the shared gene signatures are not directly linked to proliferation but to adhesion (for example, Tnc, Ly6e, Cdh3), protein phosphorylation (Melk), transcription (Id1), development (Ext1) and signal transduction (Ect2, Gpr126) (Tables 4 to 6).

**Table 5:** Correlation of mouse mammary stem-like gene expression and breast subtype signatures

High at stem-like stage	Low at stem-like stage	Unchanged
**Normal breast-like (ER-negative)**: 10 genes, none of which (0%) changed in stem-like stage		
0 genes	0 genes	10 genes

		Fhl1 – cell differentiation
		Cd36 – cell adhesion
		Itga7 – cell adhesion
		Leprotl1 – unknown
		Gpx3 – oxidation reduction
		Gpd1 – oxidation reduction
		Aoc3 – oxidation reduction
		Lpl – lipid catabolic process
		Aqp7 – transport
		Cidec – apoptosis

**Her2^+ ^(ER-negative)**: four genes, one of which (25%) changed in stem-like stage		
1 gene	0 genes	3 genes

Traf4 – regulation of apoptosis		Erbb2 – cell proliferation
		Grb7 – signal transduction
		Smarce1 – chromatin modification

**Luminal A (ER-positive, p53 mut)**: 13 genes, three of which (23%) changed in stem-like stage		
0 genes	3 genes	10 genes

	ERα – regulation of transcription	Gata3 – regulation of transcription
	Myo6 – regulation of transcription	Foxa1 – regulation of transcription
	Xbp1 – regulation of transcription	Aff3 – regulation of transcription
		Npnt – cell adhesion
		Anxa9 – cell – cell adhesion
		Gpr160 – signal transduction
		Slc39a6 – ion transport
		Tff3 – defense response
		Acadsb – lipid metabolic process
		Nat1 – metabolic process

**Luminal C (ER-positive, p53 mut)**: eight genes, six of which (75%) changed in stem-like stage		
5 genes	1 genes	2 genes

Mybl2 – regulation of transcription	Ggh – glutamine metabolic process	Ywhaz – protein targeting
Ybx1 – transcription		Sqle – oxidation reduction
Tfrc – endocytosis		
Ebna1bp2 – unknown function		
Kif23 – cell cycle		

**Basal like (ER-negative, p53 mut)**: 15 genes, nine of which (60%) changed in stem-like stage		
5 genes	4 genes	6 genes

Cdh3 – cell adhesion	Trim29 – transcription	Tnni2 – regulation of transcription
Lamc2 – cell adhesion	Slpi – serine – type endopeptidase inhibitor activity	Nfib – regulation of transcription
Krt17 – epidermis development	Galnt3 – metabolic process	Capn6 – proteolysis
Krt5 – epidermis development	Sox9 – transcription/regulation of cell proliferation	Dmd – peptide biosynthetic process
Cxcl1 – negative regulation of cell proliferation		Tgfb2 – apoptosis
		Fabp7 – regulation of cell proliferation

From Sorlie *et al*.[10]. Names and biological process (as defined by gene ontology, selected and/or abbreviated to fit table) are provided. ER, estrogen receptor.

**Table 6:** Correlation of mouse mammary stem-like gene expression and published breast tumor lung metastasis signatures

Lung metastasis signature: 14 genes, nine of which (64%) changed in stem-like stage		
High at stem-like stage (9 genes)	Low at stem-like stage (0 genes)	Unchanged (5 genes)

Id1 – regulation of transcription		Kynu – metabolic process
Tnc – cell adhesion		Man1a1 – metabolic process
Ly6e^a ^– cell surface receptor-linked signal transduction		Vcam1 – membrane to membrane docking
Ltbp1 – growth factor binding		Cxcr4 – apoptosis
Angptl4^a ^– regulation of apoptosis		Nedd9 – cell cycle/cell adhesion
Ptgs2 – regulation of cell proliferation		
Cxcl1 – negative regulation of cell proliferation		
Ereg – regulation of mitosis		
Fscn1 – cell proliferation		

From Minn *et al*. [38]. Names and biological process (as defined by gene ontology, selected and/or abbreviated to fit table) are provided. ^a^High in pre-differentiated stage only.

Our results are in line with the recent report of poorly differentiated aggressive human tumors showing an embryonic stem cell-like gene expression signature [45]. Here a core set of nine embryonic transcription regulators was found to be overexpressed at the mRNA level in many poorly differentiated tumors, and in the HC11 cells we observed four of these transcription factors to be overexpressed at the stem-like cell stage (Mybl2, Hmga1, Hmgb3, Tead4). In conclusion, the comparisons presented here show that breast cancer subtypes defined by, for example, Sorlie and colleagues [10] can be further subdivided according to stem cell-like resemblance, and comparison with both the 70-gene signature, and Sorlie's classification reveals that stem cell-like expression infers worse prognosis. We speculate that subtypes with a higher degree of stem cell-like gene expression may have a higher fraction of cancer stem cells, yielding a more aggressive cancer. Specific markers to determine whether a tumor is stem cell-like, of which we here suggest several candidates, could be important for diagnosis and treatment decisions.

Further, in the transcriptome analysis we observed a relation between mammary stem-like cell differentiation and regulation of skeletal development genes (including osteoblastic stem cell markers Spark and Spp1 [30]). In addition, both Il6, which functions as a differentiation regulator of preosteoblast cells [46], and the corresponding downstream osteoblast-specific differentiation marker Runx2 decreased when stem-like cells entered differentiation, and Ocil, a negative regulator of osteoclast differentiation, showed a robust increase at both differentiation stages. This finding may indicate why breast tumors have a preference for skeletal metastases, and these genes may have a potential as metastasis markers. Indeed, other genes implicated in bone metastasis of breast cancer cells (Ctfg, Fst and Dusp1 [47] and Adamts1 [47,48]) were altered during the differentiation of mammary stem-like cells. Expression of Adamts1 at the protein level is also shown in Figure 2d as elevated at the stem-like cell stage. In addition, lung metastasis gene expression also has an apparent parallel to stem cell-like gene expression (Table 6); lung metastasis signature genes [38]) change considerably during differentiation, most of them being elevated at the stem cell-like stage – for example, the cytokine angiopoietin-like 4, shown to prime breast cancer cells for lung metastasis [49]. The correlation of stem cell-like gene expression to metastasis signatures may in part explain the above correlation to poor prognosis.

Our approach using cross-species comparisons of murine mammary stem cell-like expression and human tumor gene expression to unlock evolutionarily conserved breast cancer–stem cell networks has provided highly concordant observations. Furthermore, this approach recently gained support, as cross-species comparisons were shown to be a powerful means of identifying essential connections [50].

### Mammary stem-like cell differentiation compared with *in vivo *mammary gland

We were interested to see whether the changes observed during stem-like cell differentiation showed any resemblance to the *in vivo *mammary gland differentiation, keeping in mind that in the mammary gland the stem cells only constitute a minority of all cells, and that their gene expression is likely to be masked by changes in other cells as well as by changes in the relative proportions of different cell types. The cellular three-dimensional structure, interaction with stroma and *in vitro *versus *in vivo *signaling, also makes the two systems very different. We compared mRNA levels of HC11 cells with 2-month-old virgin, pregnant and lactating mammary glands, where the proliferating stage could be compared with the actively proliferating pregnant mammary gland. Genes that were overexpressed at the stem cell-like stage (Birc5, Areg, Ereg, Cyclin D, Lif and Hist1h1a) all had increased expression in the pregnant glands but decreased their expression in the lactating glands (data available in Additional file 2). For the genes whose expression was low at the stem cell-like stage but was upregulated as the cells differentiated, several were also expressed at a low level in the virgin gland and were upregulated in pregnant and/or lactating gland (Expi, Ecad, Perp, Ehf, Nfat), whereas two genes (Msi2h and Mmp15) showed an opposite regulation and decreased during *in vivo *gland differentiation (Additional file 2). Although this comparison is relatively simplistic, our data indicate that stem cell-like proliferation is highest in the pregnant mammary gland whereas genes robustly expressed at terminal differentiation of HC11 cells are also highly expressed in the lactating mammary gland.

Among the nuclear receptors, we found that COUP-TFII and NURR1 decreased during differentiation of the HC11 cells and in the transition from pregnant to lactating mammary gland. ERβ and VDR, both suggested to be protective in breast cancer [8,40,51], were further induced in differentiated (lactating) mammary gland compared with virgin gland, whereas ERα – which is often increased in breast cancers – was reduced. Comparison of a published study of *in vivo *mouse mammary glands, investigating 10-week nulliparous mammary glands and 18-day pregnant glands [52], with our data on HC11 cellular differentiation shows that as many as 279 genes changed in HC11 stem-like cells were also changed in the pregnant mammary gland, further showing that there is numerous correlations between the *in vitro *and *in vivo *systems in terms of differentiation.

## Conclusions

The aim of the present study was to characterize the differentiation process of the stem cell-like HC11 cell line and to define the transcriptome of proliferating undifferentiated mammary epithelial stem cell-like cells, in relation to their differentiated counterparts. This provides a basis for research on mammary stem cells; both known and novel stem cell gene expression characteristics were found. Characterized mammary stem cell markers are highly needed, and several potentially suitable targets detected in our study will be valuable to investigate further.

We explored whether there is a link between mammary stem-like cell gene expression and that of breast cancer. The present study was performed using an *in vitro *system of mammary stem-like cell differentiation. *In vitro *systems have drawbacks, and rarely fully resemble the *in vivo *situation, but can nonetheless yield significant information. We found an interesting correlation between the pattern of stem-like cell expression and that of human breast cancer with poor prognosis, metastasis and tumor subtypes. This may indicate that some breast tumors have a high ratio of cancer stem cells, and may require specific and aggressive treatment. An amount of novel gene expression data is presented, with implications for stem cell and mammary gland development biology and breast cancer research. A scheme is provided where key differences between differentiation steps can be dissected. We conclude that HC11 cells are relevant for studying mammary stem cells, their differentiation and their relationship to breast cancer.

## Abbreviations

ER: estrogen receptor; FBS: fetal bovine serum; PBS: phosphate-buffered saline; PCR: polymerase chain reaction.

## Competing interests

The authors declare that they have no competing interests.

## Authors' contributions

CW conceived of the study and participated in the design and coordination, carried out the transcriptome studies, real-time PCR analysis, bioinformatic analysis and comparisons, and drafted the manuscript. LH participated in the design, carried out the cell cultures, mammary gland tissue dissection and immunostainings, and helped to draft the manuscript. KE performed laboratory work and helped to draft the manuscript. L-AH participated in the design of the study and helped to draft the manuscript. J-ÅG helped to draft the manuscript. All authors read and approved the final manuscript.

## Supplementary Material

Additional data file 1 Excel file containing a table that lists all differentially expressed genes detected in the microarray analysis. Gene symbol, gene name, GenBank, and changes between the different stages with corresponding statistical values are given in separate columns. Click here for file

Additional data file 2 Adobe file containing a figure that shows the real-time PCR confirmations of differentially expressed genes: confirmation of microarray results and correlating changes in *in vivo *mammary glands, of genes regulated during differentiation of HC11 cells. Click here for file

Additional data file 3 Adobe file containing a figure that shows the real-time PCR confirmations of differentially expressed genes: correlating changes in *in vivo *mammary glands, of genes regulated during differentiation of HC11 cells. Click here for file

Additional data file 4 Adobe file containing a figure that shows the real-time PCR confirmations of differentially expressed genes: expression of nuclear receptors and/or related genes increasing during HC11 mammary stem-like differentiation and correlating changes *in vivo *mammary glands. Click here for file

Additional data file 6 image file containing a figure that shows expression of vimentin analyzed by immunofluorescence in HC11 stem cell-like, pre-differentiated and differentiated cells. Click here for file

Additional data file 7 image file containing a figure that shows expression of the milk protein beta casein analyzed by immunofluorescence in HC11 stem cell-like, pre-differentiated and differentiated cells. Click here for file

Additional data file 5 Adobe file containing a figure that shows the real-time PCR confirmations of differentially expressed genes: expression of nuclear receptors and/or related genes decreasing during HC11 mammary stem-like differentiation and correlating changes *in vivo *mammary glands. Click here for file
