# Supplementary material for: Gene expression in murine mammary epithelial stem cell-like cells shows similarities to human breast cancer gene expression
Source: Breast Cancer Res. 2009 May 8;11(3):R26. doi: 10.1186/bcr2256 (PMC2716494; doi:10.1186/bcr2256)
Supplement: Additional data file 2 — Adobe file containing a figure that shows the real-time PCR confirmations of differentially expressed genes: confirmation of microarray results and correlating changes in in vivo mammary glands, of genes regulated during differentiation of HC11 cells. [file bcr2256-S2.pdf]

Supplementary Figure 1A. Confirmatory real-time PCR analysis of genes down-regulated (left panel) or up-regulated (right panel), during differentiation of HC11 cells. Ranking of the likelyhood of differential expression in the microarray analysis below. Average of three cell cultures and differentiations.

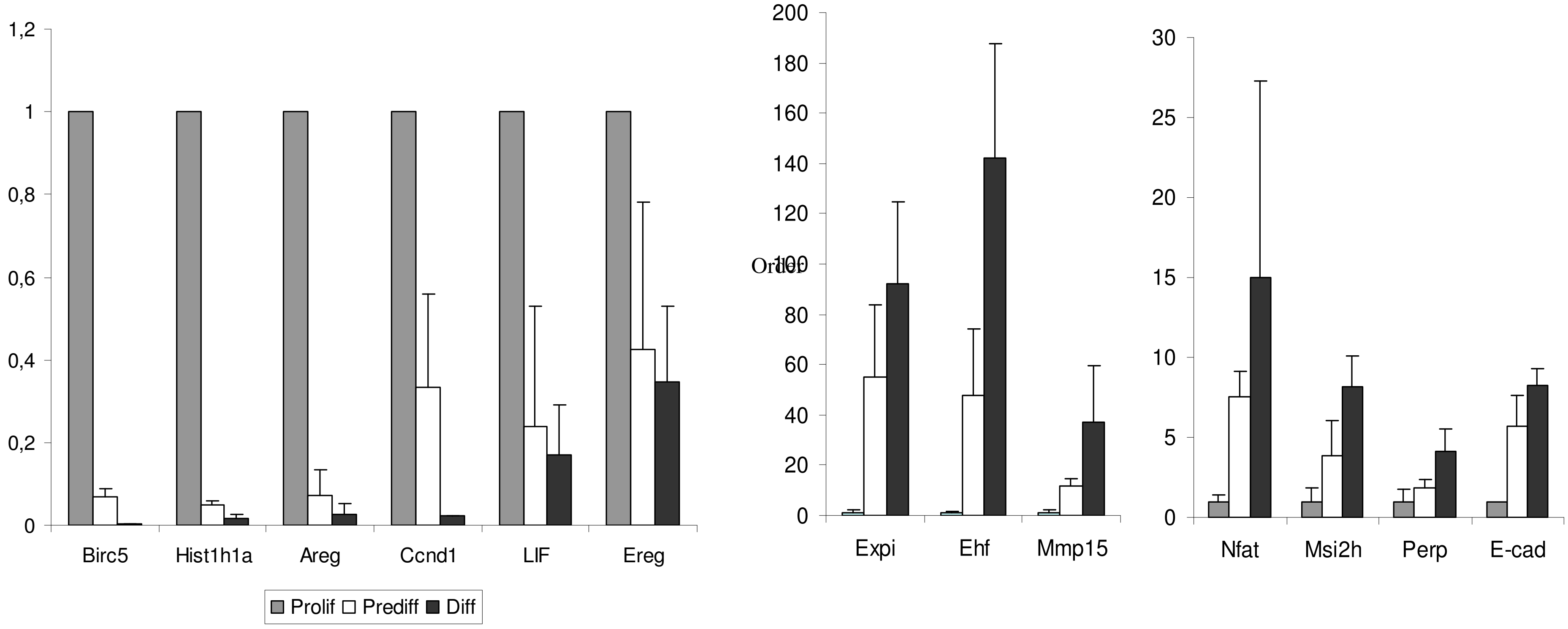

No **20** **21** **41** **13** **175** **100** **1** **7** **1000** **2086** **272** **255** **1546**

Microarray Rank Order
