# Supplementary material for: Gene expression in murine mammary epithelial stem cell-like cells shows similarities to human breast cancer gene expression
Source: Breast Cancer Res. 2009 May 8;11(3):R26. doi: 10.1186/bcr2256 (PMC2716494; doi:10.1186/bcr2256)

Supplementary Figure 1B. Correlating changes in *in vivo* mammary glands, of genes up-regulated (upper panel) or down-regulated (lower panel), respectively, during differentiation of HC11 cells. Average of 3-5 mice in each stage.

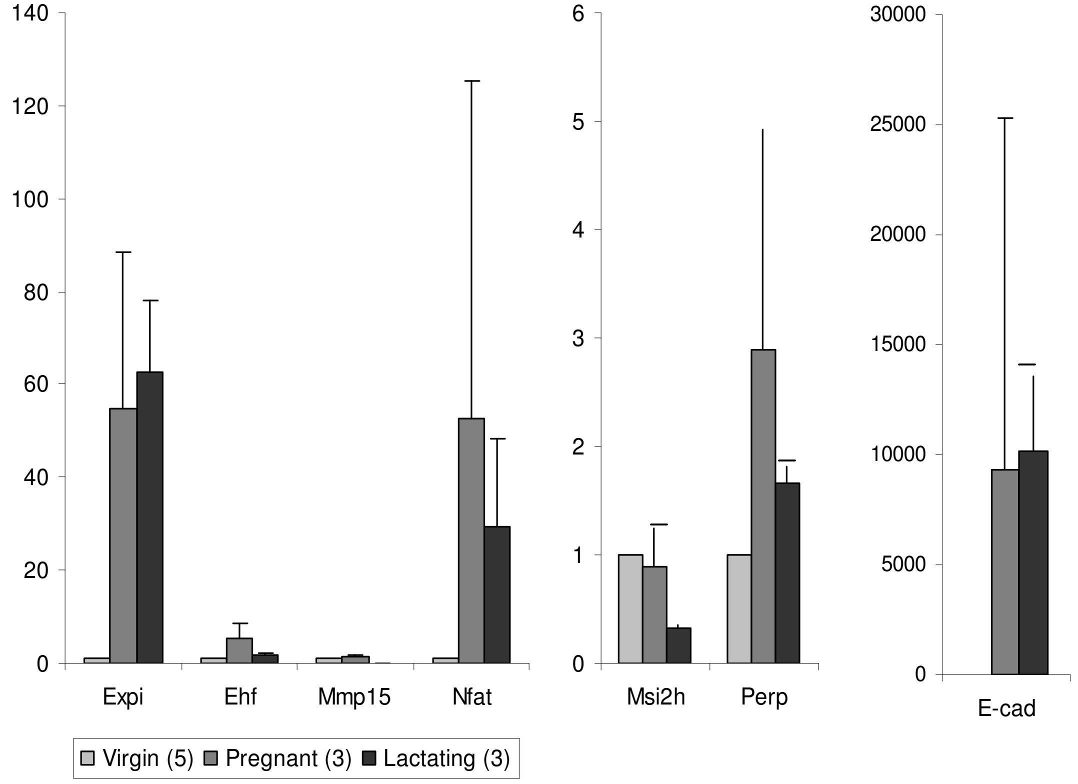

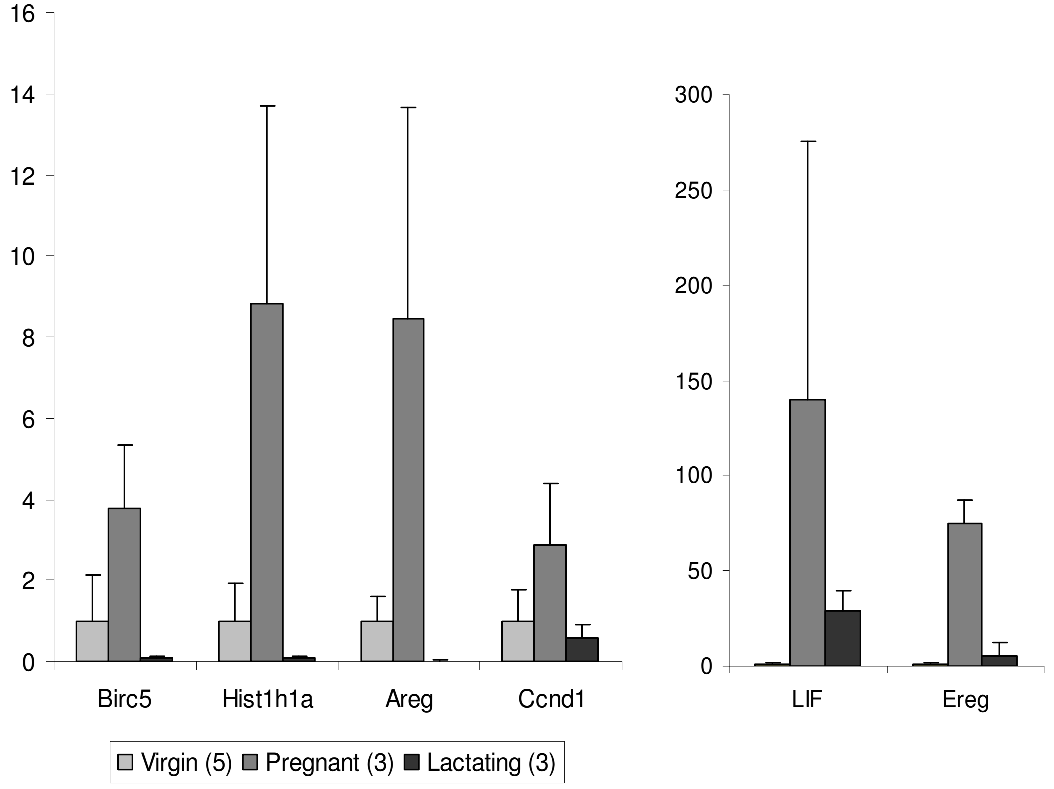

Supplement: Additional data file 3 — Adobe file containing a figure that shows the real-time PCR confirmations of differentially expressed genes: correlating changes in in vivo mammary glands, of genes regulated during differentiation of HC11 cells. [file bcr2256-S3.pdf]
