# Supplementary figures and images for: Gene expression in murine mammary epithelial stem cell-like cells shows similarities to human breast cancer gene expression
Source: Breast Cancer Res. 2009 May 8;11(3):R26. doi: 10.1186/bcr2256 (PMC2716494; doi:10.1186/bcr2256)

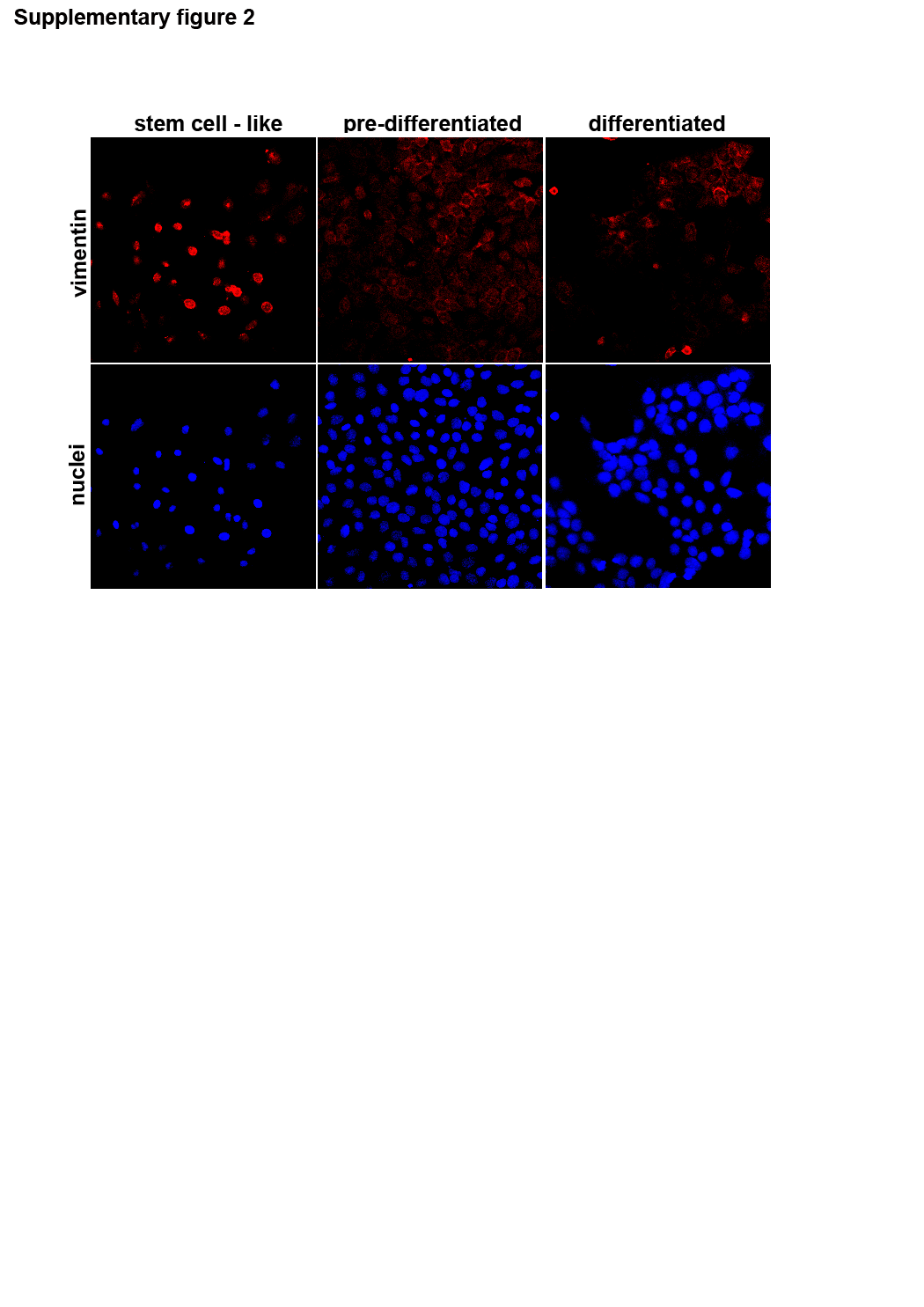

Supplement: Additional data file 6 — image file containing a figure that shows expression of vimentin analyzed by immunofluorescence in HC11 stem cell-like, pre-differentiated and differentiated cells. [file bcr2256-S6.tiff]

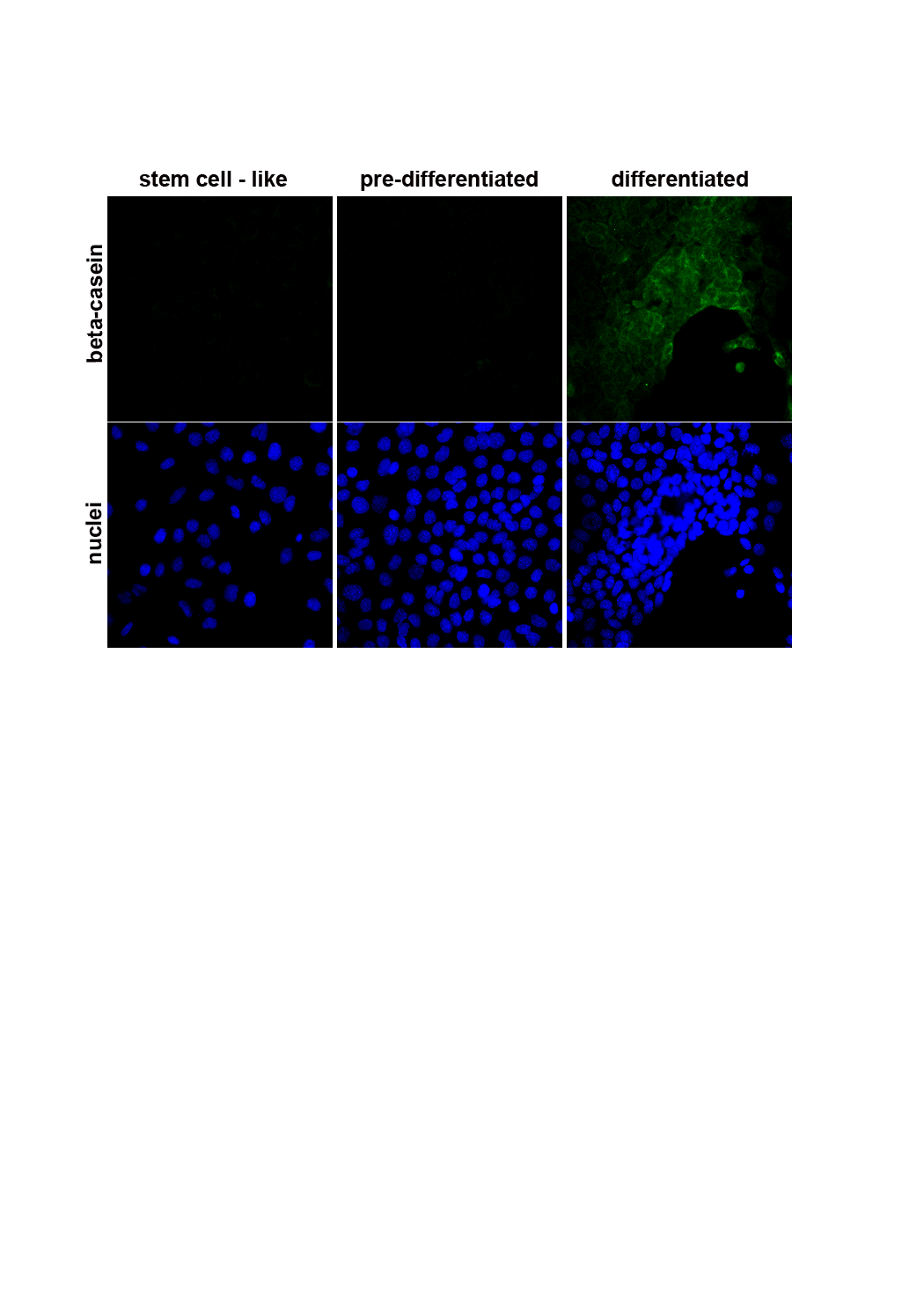

Supplement: Additional data file 7 — image file containing a figure that shows expression of the milk protein beta casein analyzed by immunofluorescence in HC11 stem cell-like, pre-differentiated and differentiated cells. [file bcr2256-S7.tiff]
