# Supplementary material for: Gene expression in murine mammary epithelial stem cell-like cells shows similarities to human breast cancer gene expression
Source: Breast Cancer Res. 2009 May 8;11(3):R26. doi: 10.1186/bcr2256 (PMC2716494; doi:10.1186/bcr2256)

Supplementary Figure 1D. Expression of nuclear receptors and/or related genes decreasing during HC11 differentiation (upper panel) and correlating changes in *in vivo* mammary glands (lower panel), respectively. Average of three independent cell cultures and differentiations; or 3-5 mice in each stage, respectively. NR4a2 (NURR1), Nr4a1(NGFIB), Nr2f2 (COUP-TFII).

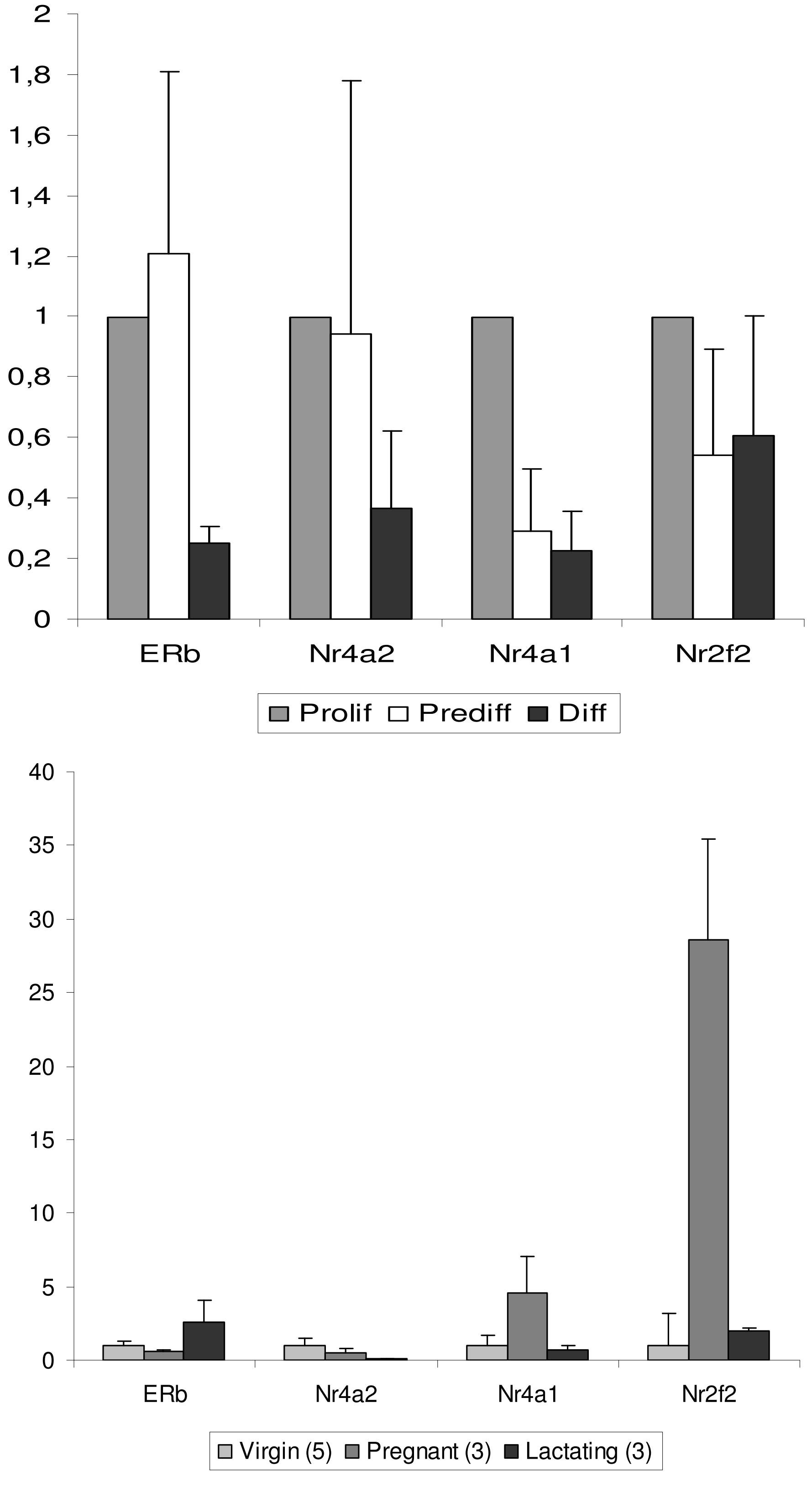

Supplement: Additional data file 5 — Adobe file containing a figure that shows the real-time PCR confirmations of differentially expressed genes: expression of nuclear receptors and/or related genes decreasing during HC11 mammary stem-like differentiation and correlating changes in vivo mammary glands. [file bcr2256-S5.pdf]
